# Context-Dependence and the Development of Push-Pull Approaches for Integrated Management of *Drosophila suzukii*

**DOI:** 10.3390/insects10120454

**Published:** 2019-12-15

**Authors:** Jeroen T. Alkema, Marcel Dicke, Bregje Wertheim

**Affiliations:** 1 Laboratory of Entomology, Wageningen University, P.O. Box 16, 6700AA Wageningen, The Netherlands; Jeroen.Alkema@wur.nl; 2 Groningen Institute of Evolutionary Life Sciences, University of Groningen, P.O. Box 11103, 9700 CC Groningen, The Netherlands

**Keywords:** behaviour, repellent, attractant, invasive pest, evolution

## Abstract

Sustainable pest control requires a systems approach, based on a thorough ecological understanding of an agro-ecosystem. Such fundamental understanding provides a basis for developing strategies to manipulate the pest’s behaviour, distribution, and population dynamics, to be employed for crop protection. This review focuses on the fundamental knowledge required for the development of an effective push-pull approach. Push-pull is a strategy to repel a pest from a crop, while attracting it toward an external location. It often relies on infochemicals (e.g., pheromones or allelochemicals) that are relevant in the ecology of the pest insect and can be exploited as lure or repellent. Importantly, responsiveness of insects to infochemicals is dependent on both the insect’s internal physiological state and external environmental conditions. This context-dependency reflects the integration of cues from different sensory modalities, the effect of mating and/or feeding status, as well as diurnal or seasonal rhythms. Furthermore, when the costs of responding to an infochemical outweigh the benefits, resistance can rapidly evolve. Here, we argue that profound knowledge on context-dependence is important for the development and implementation of push-pull approaches. We illustrate this by discussing the relevant fundamental knowledge on the invasive pest species *Drosophila suzukii* as an example.

## 1. Introduction

The aim of integrated pest management (IPM) is to design a cropping system that manages pests below the economic damage threshold [1]. Main components of IPM are biological control, host plant resistance, cultural control, and the use of infochemicals such as pheromones and repellents (behavioural control). While approaches focusing merely on pesticides aim at eradicating pests, IPM seeks to manage pests below the economic injury threshold by promoting activities that support factors regulating the pest while mitigating factors that interfere with such regulating factors [1,2,3]. Developing effective and efficient IPM strategies requires a thorough ecological understanding of the crop system. This includes, but is not restricted to, an understanding of how the pest interacts with its resources such as the crop plants, non-crop plants surrounding the crop, as well as conspecifics, competitors, and natural enemies [1]. Successfully implemented IPM strategies have been shown to result in improved resilience of the crop system, and to be financially and environmentally more beneficial than pesticide use [4,5,6]. 

### 1.1. Push-Pull Approach as Part of Integrated Pest Management (IPM)

**Figure 1:**
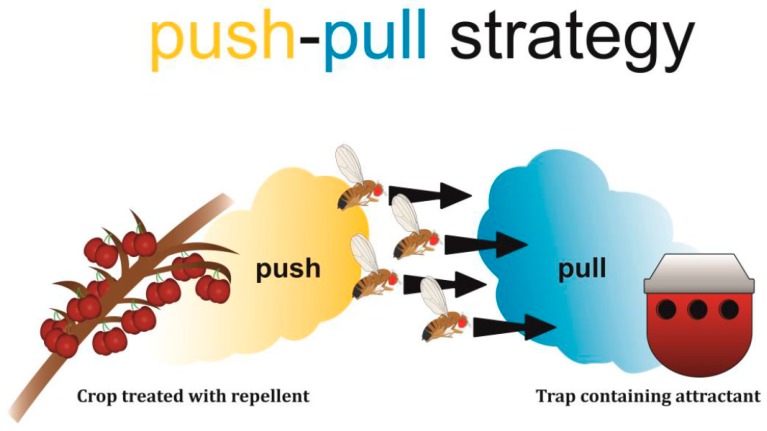
The push-pull strategy employs repellent (push) and attractive (pull) odours to deal with insect pests. Crops to be protected from a pest, drosophilid flies in this example, are treated with a repellent (yellow cloud on the left). This repellent pushes the pest away from the crop. Simultaneously, a trap containing an attractive volatile compound pulls the pest away from the crop (blue cloud on the right).

One tactic within IPM is the push-pull approach, which aims to manipulate the behaviour of pest insects using a two-pronged strategy: to push the target organism away from a crop while pulling it toward a trapping system (Figure 1) [6,7,8]. Pushing and pulling is achieved by using repellent (push) and attractant (pull) cues, usually volatiles including pheromones and allelochemicals [7]. The trap to which the insects are guided can either be a designed killing trap (e.g., a container or adhesive strip with an attractant) to capture, sequester, and kill the roaming pest insects, or a trap plant as an alternative host plant for the pest [6,7]. Candidate trap plants are highly attractive and include sub-optimal or lethal hosts that hamper the development of the pest insect, or high-quality plants that are destroyed before the pest can disperse to the crop [7,8,9]. 

The push-pull approach can form an integral part of a crop management system that prevents outbreaks; it is not suited to deal with the pest species when its population density has already reached high levels. The push system is likely to be less effective at high pest densities when there is strong competition among pest individuals, and large populations have an increased risk of evolving resistance to either the push or the pull component [7,10]. Cook, Khan, and Pickett [7] therefore proposed to apply the push-pull method before the pest species has been detected, e.g., at the start of the crop season. Thus, the push-pull approach should be a true component of the total crop management system in place [7]. Using lethal traps would reduce the spread of organisms that develop resistance to either the push or the pull element [11]. Alternatively, the population stress on the push-pull method could be relieved by reducing pest population size through natural enemies of the pest [7].

An example in which the push strategy has been very effective is the application of the host-marking pheromone of the tephritid fruit fly *Rhagoletis cerasi* in cherry orchards. Spraying of crops with the host-marking pheromone reduced tephritid infestations by 90% [10,12]. The oviposition-deterring pheromone underlying this behaviour was isolated and has been developed into an optimized blend [10,13]. However, habituation and resistance to this pheromone was observed after some time [10,14]. To avoid this, it is more effective to combine a push strategy with a pull strategy toward a lethal trap [7]. 

### 1.2. Fundamental Ecological Knowledge Required to Design Effective Push-Pull Systems

To design and implement a push-pull strategy effectively, fundamental knowledge is required [7]. This knowledge should go beyond an understanding of repellent or attractive properties. More detailed insight into the evolution of a cue, how it affects an organism, and why it is repellent or attractive will illuminate the degree to which it is susceptible to the pest evolving resistance. Once candidate infochemicals have been identified, knowledge on their context dependency is required. While a female insect may be attracted to specific chemical cues during mate searching, the female is not necessarily attracted to such pheromones once she has mated and fertilized her eggs [15,16]. In that context she may be more responsive to cues that will aid her in finding a suitable host plant for oviposition [15]. These dynamic changes in responsiveness to attractive or repellent stimuli, depending on the motivational state of the insects, have been formalized in the “Rolling Fulcrum model” [17]. Finally, it is also important to investigate how the push-pull components affect the agro-ecosystem in which they are presented. Understanding how the crops, beneficial arthropods, and non-target insects react to these stimuli is essential to develop a sustainable system.

### 1.3. Invasive Pest Drosophila suzukii

In this perspective paper, we discuss the development of a push-pull approach and how this can be optimized with fundamental knowledge on context dependency. We first briefly describe the various classes of infochemicals that can be exploited in a push-pull approach, and their evolution in pest insects. Then we discuss the concept of context dependence for behavioural manipulation, and how this matters in pest management strategies. Next, we present the relevant fundamental knowledge on the invasive pest species *Drosophila suzukii* as an example to illustrate the added value of considering context dependency. Finally, we provide recommendations for how a push-pull approach may be implemented in the context of IPM of *D. suzukii*. 

## 2. Infochemicals

Organisms need to make decisions such as where to search for food, which food source to accept, and whom to accept as a sexual partner. Such decisions should be based on information, and chemicals provide a rich source of information. Chemicals that convey information (infochemicals) can affect both the emitter and the organisms that perceive them [18,19]. Infochemicals can be volatile and function in information conveyance over short- to long distances [20,21], or they can be non-volatile, being only perceived through direct contact or gustation [20]. There is a rich array of sources of infochemicals, as well as a rich array of consequences in ecological interactions [22,23,24].

Two classes of infochemicals can be distinguished. First, allelochemicals are infochemicals that mediate interactions between members of different species [18,19]. The release of these chemicals can enhance the fitness of the sender [18,19], e.g., plants releasing volatiles after an attack by herbivorous insects that attract the natural enemies of these insects [25], or volatiles emitted by flowers to attract pollinators [26]. Alternatively, allelochemicals can be released without serving a particular function to the sender, but may still reveal relevant information to receivers [18,19], such as volatile compounds emitted by waste products or frass that may inadvertently reveal the location of prey. The supply of information through allelochemicals is abundant, and organisms are under selection to exploit the subset of information that is relevant in their ecology [24].

The second class of infochemicals comprises pheromones, which mediate interactions between conspecifics [18,19]. Pheromones mediate a multitude of behaviours in intraspecific interactions, e.g., alarming conspecifics [18,27], attracting sexual partners [18,28,29,30], or providing information on the location [31,32] or quality [33] of a resource. Pheromones are usually produced in designated glands, and the biosynthesis may require specific nutrients [34]. Pheromones often convey specific information that may have a function for the emitter and receiver [18]. When the conveying of this information results in enhanced fitness for the emitter and the receiver, the emitter will be under selection to invest in pheromone production and the receiver in pheromone perception.

The best-known pheromones are sex pheromones. These pheromones inform individuals of the opposite sex about the presence and location of the emitter. Sex pheromones are typically released by males or females that are reproductively mature, and they attract members of the opposite sex that are searching for a mate. In intersexual communication sex pheromones can be indicators of the quality of the emitter and, consequently, receivers may use these pheromones selectively in their mate-choice [35]. Additionally, sex pheromones are often highly species-specific and thus facilitate species discrimination during mate finding and mate choice [36].

Aggregation pheromones inform conspecifics of both sexes about the presence of conspecifics [37] and/or the presence of a breeding or feeding site [31]. The receivers that are attracted to these pheromones may achieve higher survival for themselves and/or their offspring. Emitting aggregation pheromones is subject to positive selection if the emitter is also subject to these advantages. The same compound may also serve multiple functions, e.g., as an aggregation- as well as a sex pheromone [32]. It may serve as an attractant in combination with food odours to an insect searching for food or a breeding site, and once the insects have aggregated, the same compound may serve as a sex pheromone [37].

Some insects deposit host-marking pheromones to signal to other females that the host is occupied or infested. When this pheromone makes the occupied host unattractive to conspecifics, the pheromone can be considered an oviposition deterrent [38]. For instance, tephritid fruit flies that have oviposited in a fruit subsequently mark the fruit by depositing a pheromone. This renders the fruit less acceptable to conspecific flies. Consequently, it reduces competition for the emitter’s offspring [39]. These host-marking pheromones evolved in different orders of insects and in different ecological settings, and generally seem to act as close-range deterrents [38,40].

An infochemical that benefits the interactions among conspecifics may bear costs to the producer when it mediates the attraction of other species as well [24]. Predators and parasitoids excel in exploiting sex- or aggregation pheromones to localize their prey or hosts. Parasitoids lay their eggs in or on/near other insects, and they can exploit the pheromones emitted by these insects to find their hosts. Because the infochemicals are thus detrimental to the emitter in this particular interaction, this imposes selection on the emitter to reduce its emission. However, when these same compounds are beneficial in intraspecific communication, this imposes positive selection on their emission. As long as the benefits of an infochemical outweigh the costs, the infochemical will persist in the biology of the species [41]. 

In conclusion, organisms are exposed to a wide variety of infochemicals in the environment, both emitted by conspecifics and heterospecifics. These infochemicals, alone or in combinations, provide an organism with information that can be used to make decisions to maximize its reproductive fitness in a heterogeneous environment. Knowledge of how organisms respond to infochemicals may provide the basis of behavioural manipulation, e.g., with the aim to manage their population size in the context of pest control [42,43,44]. Importantly, though, infochemicals function in a context-specific manner, both in conspecific and interspecific interactions. 

## 3. Context-Dependence

Individuals are exposed to various environmental cues and the sensory information of these cues is integrated in the central nervous system [29,45]. Moreover, the physiological state of an individual influences its behavioural responses to these environmental cues [29,45]. Early life experiences, learning, and habituation can further modulate the behavioural responses to environmental cues [46]. As a consequence, an organism may display certain behaviours in response to a stimulus, yet these behaviours may differ when the organism is tested in another environment or when the organism is in a different physiological condition [47]. This context-dependent aspect of behaviour is important to understand how and when an organism responds in the field to cues, where it is exposed to a broader range of stimuli than under laboratory conditions. 

Behavioural responses are often plastic and dependent on the physiological state of the organisms. As earlier mentioned, virgin female insects have a different physiology and motivation than mated females, and can be more responsive to cues that present them with opportunities to copulate, while mated females may be more attracted to cues that indicate the presence of feeding or oviposition sites [15,16]. Moreover, the sexes and the different developmental stages may differ in their behaviours toward environmental and social cues. For example, milkweed bug nymphs and young adults differ from older adults in their responsiveness to social cues [48]. Additionally, some insects show strong phenotypic plasticity, which can also drastically modify their responsiveness to environmental and social cues. A clear example is the desert locust, *Schistocerca gregaria*, which exhibits striking differences in morphology, physiology, behaviour, and chemical ecology between the solitary and gregarious phase [49]. 

The composition of the infochemical blend presented to insects also matters. Webster et al. [50] tested 15 compounds of the black bean aphid’s (*Aphis fabae*) host plant *Vicia faba*. Nine of the host volatiles induced negative responses when presented individually to the aphids, while the blend of these nine compounds, at the same concentrations as when tested individually, attracted the aphids [50]. The ratio among individual compounds can lead to synergy and increases the attractiveness of the blend beyond that of the sum of its components. In sex pheromones, the ratio of the different components in the blend can provide species-specificity, which can be crucially important for the effectiveness as attractant or repellent [36]. 

Some infochemicals are only attractive when accompanied by other environmental cues [51], such as aggregation pheromones that require the simultaneous presence of food odours. For instance, the aggregation pheromone of the fruit fly *Drosophila melanogaster*, 11-*cis*-vaccenyl acetate, only acts as long-range attractant in the presence of yeast-produced odours [31]. This same compound also acts in several other social (intraspecific) interactions in *D. melanogaster*, and its diverse effects are modulated by environmental odours [52]. Moreover, volatile cues may require an interaction with other sensory modalities, such as visual stimuli [53]. 

Context also has a temporal component. Diurnal and seasonal rhythms are prevalent in both behaviour and physiology, and can have important effects on the responsiveness to environmental cues, e.g., restricting responsiveness to social cues to particular periods of the day or season [54,55]. Moreover, both experience and learning may influence the behavioural responses to environmental cues later in life. For instance, *Daphnia magna* becomes more negatively phototactic when it is exposed to predator-produced odours (fish kairomones) during its development [56]. Also social experiences can modify responses to environmental cues. For example, in *D. melanogaster* the presence of either food odours or social odours (pheromones) reduced the mating latency in virgin females and increased the number of copulations they engaged in within 24 h, while depriving flies of social interactions during early life (by rearing them in isolation) prolonged the latency and reduced the frequency of mating [57,58]. Furthermore, when a *D. melanogaster* female observes courting conspecifics, this alters her willingness to mate with a male based on its success in courting another female [59]. Thus, various aspects of behaviours are dependent on context and experience.

## 4. *Drosophila suzukii*—A New Invasive Pest

In the past decade a new invasive pest has emerged, the Spotted Wing Drosophila or *Drosophila suzukii*, that originates from Asia. In Europe, *D. suzukii* was first recorded in Spain in 2008, and retrospectively was discovered to have been present in Italy at the same time [60]. Within a year the fly had spread throughout these countries and had moved into France [60]. In 2009, the first crop damage occurred on cultivated berries [60,61]. By 2012 the species had spread throughout other European countries including Switzerland, Slovenia, Croatia, Austria, Germany, Belgium [60], and the Netherlands [62,63,64]. While *D. suzukii* settled in Spain and Italy in 2008, it did so too in California [65,66]. Currently, it has been reported across the United States [66,67,68] and started invading Canada [66,68,69]. In Brazil, *D. suzukii* was first recorded in 2013 and it has spread throughout the South American continent since then [70]. Future invasion of Australia and Africa has been predicted [71]. It thus appears that this fly is becoming a globally established pest species.

*Drosophila suzukii* can migrate over substantial distances and infest many different field crops. In infested crops, up to 75–80% of the fruits may contain eggs and larvae [61,72]. The larvae develop and feed inside the fruit, causing the flesh of the fruit to turn brown and soft. Once the integrity of the fruit is compromised by *D. suzukii* oviposition and feeding, it also becomes accessible for other pest insects. Additionally, the damage can provide an entry site for infection by fungal and bacterial pathogens. *Drosophila suzukii* oviposits in fruits of a variety of commercial crops, including strawberries, blackberries, blueberries, cherries, grapes, nectarines, peaches, plums, and raspberries [72,73]. Female *D. suzukii* have a serrated ovipositor which allows them to lay their eggs under the skin of soft fruits [68]. This capacity to lay eggs in soft-skinned fruits has led to substantial economic damage in the fruit industry [73,74]. Female *D. suzukii* can lay up to 400 eggs, spread over many fruits and there can be up to 10–15 generations per year. Population densities build up over the course of the fruiting season, from low densities from January to May, until massive numbers in September to November [65,75,76,77]. 

Control of *D. suzukii* with synthetic pesticides can be effective, but it is inefficient and harmful to the environment [78]. Moreover, resistance of *D. suzukii* to pesticides is emerging [79]. Ovipositing *D. suzukii* females are difficult to detect and cause only little initial damage, making it difficult to discover the infestation early enough to respond effectively. Once the larvae start feeding inside the fruit they are protected from pesticides that are sprayed on the fruit [80]. Therefore, commercial soft fruit growers are now advised to repeatedly apply broad-spectrum insecticides in a prophylactic manner, until refined management practices are in place [81]. The use of broad-spectrum insecticides is well-known to come with environmental problems because of the e.g., side effects on non-target species such as pollinators and entomophagous arthropods and accumulation in groundwater reservoirs. Moreover, *D. suzukii* larvae are resistant to most of the pesticides that are legally approved for use in fruticulture. 

Thus, an IPM approach is needed to provide fruit growers with a sustainable alternative to manage *D. suzukii.* This can include monitoring of the pest, using resistant crop varieties, promoting natural enemies of *D. suzukii,* as well as designing a cropping system that prevents pest outbreaks. Unfortunately, biological control methods such as the release of natural enemies have yet to show effective results for controlling *D. suzukii*. There are no species identified in the invaded area that can effectively control the *D. suzukii* population in crops. Additionally, effective biological control and crop protection for this particular pest species requires a strategy that can be applied in the various crops and which targets a common aspect of the fly’s biology. The chemical ecology of *D. suzukii* provides a number of key targets for this. Development of a push-pull strategy may therefore be a particularly promising strategy for the control of this devastating pest species. To develop and implement an effective push-pull approach, we first need to understand the behaviour and chemical ecology of *D. suzukii*.

## 5. Chemical Ecology of *Drosophila*

Most of what we know on infochemicals in the biology of *Drosophila* species comes from research on the chemical ecology of *D. melanogaster*. This species produces aggregation and sex pheromones, which both function in long-distance and short-range communication [31,82]. In addition, *D. melanogaster* is strongly attracted to odours of yeast and fermentation [83]. Many gene families that code for olfactory receptors (Odorant Receptors and Ionotropic Receptors) or taste receptors (Gustatory Receptors) have been characterized, including the developmental stage or organs in which they are expressed [84,85]. Also the neuronal circuitry for sensing and perceiving olfactory cues has been extensively investigated (e.g., [86,87,88]).

In *D. melanogaster*, males produce the aggregation pheromone 11-*cis*-vaccenyl acetate (cVA). This pheromone attracts both males and females when it is combined with food-related odours [31,89,90]. The males transfer the pheromone to females during mating [91], which makes the females less attractive to other males [92,93]. Both males and mated females release the pheromone, but females do not produce cVA themselves [91,93,94]. cVA is attractive to males, mated females, and virgin females [31,33,89,94]. In the aggregations that form on feeding and breeding sites the flies mate and the females deposit their eggs [95], which results in an aggregated distribution of eggs and larvae [90]. 

The aggregation pheromone of *D. melanogaster* is also attractive to other *Drosophila* species. Closely related *Drosophila* species resemble each other in aggregation pheromone composition, suggesting these pheromones are not species-specific [90,96,97]. The long-range attraction to the pheromone and the consequential communal oviposition benefits larval resource exploitation and survival. Larvae are engaged in interference competition with filamentous fungi that also develop on the breeding sites, and the larvae experience enhanced survival at intermediate densities, while they also experience associated costs in terms of increased food competition at high densities [90,97,98,99]. The combined effects of these intra- and interspecific interactions and the odour-guided dispersal can determine whether or not fruit fly populations can establish and persist [100]. 

Sex pheromones in *D. melanogaster* consist of cuticular hydrocarbons [92,93]. The flies produce a rich palette of different hydrocarbons that differ among the sexes and can change upon mating, feeding, and social context [101,102,103]. In females, the expression of cuticular hydrocarbons reduces the likelihood of being courted by males from other species [93,104]. Likewise, males produce 7-tricosene which has been suggested to inhibit courtship by other males [93]. The cuticular hydrocarbons are produced in specialized cells on the tergites of adult flies, the oenocytes, and ablating these cells shows that the cuticular hydrocarbons regulate both species and sexual recognition [105]. The neuronal circuitry for the detection and integration of pheromonal information has been studied in detail in *D. melanogaster* (reviewed in [106]). 

### 5.1. Drosophila suzukii

The biology of *D. suzukii* appears to differ in important aspects from that of closely related drosophilids. Its serrated ovipositor enabled it to colonize a new niche to lay its eggs in undamaged, ripening, and ripe fruits, rather than the overripe and rotting stages used by other drosophilids. Karageorgi et al. [107] proposed a five-stage evolutionary mechanism through which *D. suzukii* has been able to occupy this new niche, including altering their oviposition site preference [107,108]. The chemical ecology of *D. suzukii* received ample interest in recent years [109]. Yet, also its general biology is substantially different, including a different pace of living (e.g., reaching the peak of reproduction at a later age, and being relatively long-lived with sustained egg production) [110], and more striking phenotypic plasticity in response to seasonal cues [111]. Here, we will discuss the altered behavioural responses to substrate and social odours in the context of both the mechanisms and the evolutionary drivers of the behaviours.

First, *D. suzukii* are more sensitive to volatiles that are associated with the ripening of fruits, as shown by electrophysiological assessments for different sensilla and in behavioural assays. They detect the presence of fruits in earlier ripening stages than *D. melanogaster*, and perceive components of the changing composition of the fruit odours [112]. They are also attracted toward leaf volatiles, which may explain their residing in the plant canopy during the early developmental stages of fruits [112,113]. Correspondingly, for oviposition *D. suzukii* strongly prefers ripening fruits, whereas *D. melanogaster* and its closer relatives strongly prefer rotting fruits [107].

Yet, while it colonized the new niche, some aspects of olfactory perception and behaviour seem to be conserved. For example, it has retained avoidance of some repellents such as DEET (*N,N*-Diethyl-meta-toluamide) and fungal rot odours [72,112,114,115,116,117]. Moreover, despite its olfactory specialization on cues derived from earlier ripening fruit stages and leaf odours, yeast and fermentation odours are still highly attractive to *D. suzukii* [72,112,114]. When given a choice between odours of ripe and overripe or rotten fruits, *D. suzukii* was more attracted to the odours of overripe and rotten stages than to ripening stages, similar to most *Drosophila* species of the *melanogaster* clade [107,112]. Thus, while the sensory perception and behavioural response of *D. suzukii* toward fruit odours has changed for oviposition, it has retained its strong attraction to fermentation odours.

This brings to light an interesting paradox. *Drosophila melanogaster* larvae feed on yeast to obtain part of the essential nutrients for their development [118,119], and females also increase the rate of oogenesis when they have access to yeast [120]. This is an obligate dependence, albeit not a specific one, as *Drosophila* can obtain proteins and essential nutrients from feeding on a wide range of microbes [121]. Presumably, *D. suzukii* adults also require yeasts as a nutritional source for themselves and their offspring. Ripe and undamaged fruits likely do not contain them in high abundance, whereas fermenting and rotten fruits do. The attraction of adult *D. suzukii* to overripe fruits may therefore constitute a retained dependency on yeasts, for egg-production and/or for acquiring the yeasts that the larvae depend on during their development. The fruit collapse and brown rot that is often associated with the infestation by *D. suzukii* is often interpreted as higher susceptibility of punctured fruits to microbial infestation. Alternatively, it could indicate that females inoculate their breeding sites during oviposition to enhance the nutritional content for their offspring [122]. When the females are still largely dependent on yeast and other microbes, either for their egg production or for larval development, this can be exploited in the development of a pull strategy. Cha and colleagues have developed a four-component blend, based on the attractiveness of red wine and vinegar, to attract *D. suzukii* [123,124,125]. To be a useful element in a push-pull approach, however, the specificity of attraction is important.

*Drosophila suzukii* show an altered responsiveness to aggregation pheromones of close relatives. This is not due to a lack of perception, but it may reflect different selection pressures that operate in their new niche. The sensory neurons and associated neural system to perceive and react to the aggregation pheromone cVA are conserved in *D. suzukii,* even when it does not produce the pheromone itself [126]. However, rather than being attracted to cVA, *D. suzukii* avoids this compound [126]. This indicates that although the sensory mechanisms underlying its perception are preserved in *D. suzukii*, the behavioural response is altered. Additionally, *D. suzukii* seems to be more solitary in its egg-laying behaviour, with fruits typically only containing the eggs of a single or few *D. suzukii* females [127]. Both could be due to the different nutritional environment for developing larvae in ripening fruits. The nutritional quantity of ripening fruits, in terms of available sugars from the fruits and proteins from microbial growth, is likely to be reduced compared to overripe and rotten fruits [128,129]. Additionally, larval competition with filamentous fungi is also likely reduced, as these fungi typically do not have access to intact fruits. Fungi can secondarily infest fruits after *D. suzukii* females puncture the skin during oviposition [130]. However, in that case the *D. suzukii* larvae would have a head start. Thus, the altered conditions in ripening fruit may not accommodate, nor require, communal egg-laying in *D. suzukii*. In contrast, it is conceivable that *D. suzukii* adapted the cooperative host-marking mechanism found in *D. melanagoster* [33] to create isolated oviposition sites. This marking would then constitute a deterrent host-marking pheromone. Either of these infochemicals, i.e., the ancestral aggregation pheromone cVA, a possible host-marking pheromone, or fungal odours could be considered candidate elements for developing a push strategy.

### 5.2. Context Dependence in D. suzukii

Individuals in pest populations may represent different phenotypes with different responses to environmental characteristics. Knowledge on this is important for the development of management strategies for *D. suzukii*. The physiological state of the fly may determine its responsiveness to attractants and repellents [131]. Additionally, these attractants and repellents may only serve as such in the contexts in which they have been tested, while they may have a different effect when applied to crops with dissimilar contextual cues. A clear example is the attraction of *D. suzukii* females to yeast and fermentation odours, which is altered by the physiological state of the flies and the setting in which it is tested; these odours are strongly attractive in laboratory settings and in field monitoring traps, but they do not effectively capture gravid females in the field that prefer non-fermenting fruits for oviposition [132]. Thus, when developing traps for monitoring the presence of *D. suzukii*, a blend to resemble fermentation products may be very suitable. However, when developing traps that can compete with the actual crop, to lure egg-laying individuals away from the developing fruits, a very different blend may be more effective. Indeed females captured on ripe fruits have ovaries containing greater numbers of eggs at a more mature state than in traps containing fermentation baits [133]. Likewise, traps with fermentation baits were more effective, i.e., captured a higher percentage, of released starved flies and virgin females than flies that were fed or mated [131,132]. Thus, taking both the individual state and the environmental context into account when developing IPM can improve its success. 

Laboratory studies on *D. suzukii* have demonstrated that the flies have different levels of movement activity depending on the time of day [134]. Furthermore, the flies mate more frequently during the first 30 min of their experimentally induced day than during the remainder of the day [117]. Oviposition on the other hand is found to occur predominantly at the end of the day [134]. These temporal patterns may influence the responses to environmental stimuli. Likewise, experience may influence behavioural responses to environmental cues. Mated *D. suzukii* females are less attracted to yeast than virgins within the first hour of the experiment, although they did attain similar percentages of capture after four hours of testing [132].

Apart from the diurnal rhythms, *D. suzukii* also has a strong seasonal phenology with pronounced phenotypic plasticity. Under the influence of both photoperiod and temperature, *D. suzukii* develops as winter or summer morphs [111]. These phenotypes are markedly different, both in morphology and physiology, with winter morphs being larger, darker, more cold tolerant, and often in a state of (reversible) reproductive dormancy [111,135]. The winter morphs are predominantly produced in autumn, and the flies overwinter as adults. These winter morphs start reproducing in early spring on non-crop fruits, such as the berries in ornamental and forest plants, before moving into orchards when the first fruit crops start developing [77]. The summer morphs are produced throughout spring and summer, and these flies tend to have a higher reproductive output, and typically have access to a different range of fruits for oviposition [77,113,136]. Thus, the two morphs represent different physiological states and they also exhibit different behavioural responses to environmental and social stimuli [131,137]. Therefore, a push-pull strategy effective against one of the morphs may be less effective against the other morph. 

## 6. What Is Needed for Development of Successful IPM of *D. suzukii*?

So far, most methods to control *D. suzukii* are investigated in isolation. No single method has been developed that is effective by itself [109]. In the context of IPM, however, a combination of methods that have additive or synergistic effects may be applied that could result in effective management of *D. suzukii.* To develop an IPM approach to manage *D. suzukii* requires assimilation of fundamental understanding of its physiology and behaviour. In addition, field studies are imperative to assess the effect size of any (combination of) management approaches in an agro-ecosystem. 

### 6.1. Push-Pull

The push-pull strategy has been successfully applied to manage other pest species [4,10,12,138]. Such a method depends on designing a push and a pull component that are effectively altering the behaviour of the pest to the extent that the pest population in the crop remains below the economic injury level [1,7,139]. The push-pull strategy is especially effective for low pest densities [7]. The development of a push-pull strategy needs to account for the context-dependent issues as discussed above, before it can be effectively implemented. Furthermore, after an organism has located a resource through volatile infochemicals, other sensory modalities such as taste affect its behaviour [140]. Thus, although some repellents may be effective while the pest is airborne, the fly is likely to use other cues upon landing. The addition of a non-volatile deterrent may reduce this behaviour, especially when a proper pull element is implemented. Providing the pest with an alternative, i.e., the pull component, is important to reduce the risk of the pest developing insensitivity against the push component [7]. Various elements of the chemical ecology of *D. suzukii* are being unraveled [109]. However, major challenges include the development of cues with a considerable degree of specificity, especially for the pull component. Dedicated design of blend composition may aid in improving trapping systems.

### 6.2. Additional Density Reduction Methods

While a push-pull approach can be applied for crop protection, it may not sufficiently suppress the pest population density. This could be alleviated by the promotion or release of natural enemies of *D. suzukii,* such as predatory bugs, beetles and earwigs, larval and pupal parasitoids, entomopathogenic nematodes, fungi, viruses, and bacteria. None of the native parasitoid species in Europe and North America seems to be effective at reducing *D. suzukii* population densities (reviewed in [141]). Classical biological control, i.e., importation of a natural enemy from the region of origin of the pest, may be an alternative. The parasitoids *Asobara japonica, Leptopilina japonica*, and *Ganaspis* cf. *brasiliensis* may be good candidates [142,143,144]. For this option, however, various regulations need to be met with, including the Nagoya protocol and phytosanitary regulations for import and release of biocontrol agents to avoid unwanted effects on non-target organisms [145,146,147]. Alternatively, local natural enemy species may be improved by exploiting existing genetic variation within and between species, for example through selective breeding approaches [141,148]. Lee and Vilcinskas [149] found that viruses known to infect *D. melanogaster* are even more effective at infecting *D. suzukii.* Hübner et al. [150] identified entomopathogenic nematodes, which could attack *Drosophila* larvae after they have infested fruits in contact with the soil, and potentially even in fruits still attached to the plant. Unfortunately these nematodes cannot survive exposure to UV light [150]. They could possibly be employed in the soil to maintain crop hygiene. In IPM, crop diversification may be employed to manage pests, e.g., by providing suitable habitat for a diverse range of (generalist) natural enemies of pests [151]. However, the hidden feeding niche of *D. suzukii* larvae poses an extra challenge.

A different approach for density reduction is to hamper population growth by impairing reproduction. The sterile insect technique (SIT) employs the release of sterilized males to compete with wild males [152]. For this method to be effective, the relative competitiveness of sterile males should be comparable with that of fertile males, and ideally, females would not mate with multiple males [153]. Unfortunately, the latter does not seem to hold for *D. suzukii*. Similarly, the incompatibility insect technique (IIT) is being explored, where males of *D. suzukii* are infected with a strain of *Wolbachia* that could induce cytoplasmatic incompatibility (CI) in wild females, resulting in post-mating sterility of the female [154]. However, although different strains of *Wolbachia* exist and have been identified in *D. suzukii*, CI does not appear to be a major effect of these existing *Wolbachia* strains. 

### 6.3. Additional Methods Under Investigation

Bait sprays, consisting of edible bait contaminated with pesticides, are also being developed, to reduce the amount of pesticide needed in pest management. The effective doses for orally ingested pesticides can be drastically lower than for contact pesticides, while the effectiveness can be much higher [155,156]. Edible coatings can reduce oviposition and survivorship of immature *D. suzukii* [157]. Exclusion methods, such as enclosing the entire crops under fine netting or moving the crops into greenhouses, appears to be fairly effective, especially when additional measures are taken to eradicate any stray *D. suzukii* that breaches the enclosure [158]. Sanitary measures (e.g., removal of infested or fallen fruits) are also highly important to retard the build-up of the pest population in the area and to avoid outbreaks. 

## 7. Conclusions

A decade of research on methods to control the new global pest *D. suzukii* has shown that a silver bullet is unlikely to exist. Rather than controlling this pest by a silver bullet, a combinatorial management strategy seems to hold more promise. To manage the invasive pest *D. suzukii*, we propose that IPM is required. A push-pull strategy could form an important part of this approach. For the development and implementation of an effective combination of push and pull elements, however, we need to take into account the motivational state of flies present in the crops. Within the crops, it should focus on deterring ovipositing females away from developing fruits, as manipulating their distribution and behaviour is critical for crop protection. The crops should be protected with an olfactory push element to repel airborne flies, preferably complemented with a contact-based oviposition deterrent. Alternatively, the contact-based oviposition deterrents could be replaced with edible wax coatings. Within the crops, the pull element should thus also focus on ovipositing females, to provide an attractive alternative for these flies. For this, fermentation-based lures may not be optimal, as gravid females prefer to oviposit on ripening fruits, not overripe or rotten fruits. Conversely, trapping elements implemented outside of the crops and/or early in the season for monitoring purposes should focus on the motivational state of the flies. For these flies environmental stimuli associated with foraging and mating may be more attractive, and fermentation-products could thus be very effective for monitoring traps. Moreover, the timing and design of these strategies could be adjusted to account for differences between summer and winter morphs.

The push-pull strategy by itself is not likely to be sufficient and should therefore be supplemented with other pest management methods in order to strengthen it. Ideally, IPM should be effective in managing *D. suzukii* populations early in spring, before the fly establishes in the crops, as this could squelch the dramatic increase in population growth over the fruiting season. The release of natural enemies and exclusion methods can mitigate the density-related limitations of the push-pull strategy. Imperative to the whole strategy is proper hygiene, where any infested or unused fruit is removed from the crop in order to reduce breeding sites. In this context, some crops may be more amenable for setting up IPM than others. For instance, in strawberry production, fruits are harvested continuously, whereas in cherry production the fruits all remain on the trees until complete harvest. Also, the type of non-crop plants in the field margins may be an important element in terms of being more attractive to the crop or attracting flies from the surrounding landscape.

The importance of taking context dependence in consideration for developing IPM strategies against *D. suzukii* should be stressed, but clearly, this is not restricted to this particular invasive pest. A thorough understanding of context dependence could enhance the efficacy of IPM strategies against any pest species. IPM relies on understanding the vulnerabilities of the pest organism, and the details of its threats to the agro-ecosystem. It is certainly not new to highlight that the responsiveness of insects to cues is not constant (e.g., [17]), and much applied research is devoted to identifying exactly under which (internal and external) conditions pest insects respond to lures or deterrents. Yet, we argue that we need to more actively and explicitly incorporate fundamental knowledge on motivational states and evolutionary context for the tailored development of IPM strategies. Whether an organism responds to a cue depends on various internal and external factors. During its decision-making, the organism has access to two types of information: private and public [59,159]. Private information is information available only to the individual, obtained through interactions with its environment, such as the number of partners the female has mated with, its own feeding status and the number of matured eggs it carries [160]. Conversely, public information is available to any organism in the environment that is able to perceive it. In the development of a push-pull strategy, we need to be aware of the constraints and limitations that private information can place on the responsiveness to public information, which we can employ to manipulate pest insect behaviour. Moreover, detailed knowledge on context-dependence for both internal physiological state and external environmental conditions also allows for the development of the most appropriate set of approaches in IPM to target the vulnerabilities and threats of the pest. Developing an IPM approach is explicitly a process that assembles elements that by themselves may not yield sufficient resilience in the crop. In the case of *D. suzukii* we deal with a novel pest that emerged in a short period of time on a global scale. Research to understand the biology of this fly has grown rapidly in the past years, but many approaches aim at a silver bullet. This pest seems specifically a pest that requires concerted efforts in building an integrated pest management system. If successful this may stimulate a systems approach to the management of other pests as well, and thus contribute to a wider positive development of pest control strategies that are more sustainable.
